# Soil test phosphorus and cumulative phosphorus budgets in fertilized grassland

**DOI:** 10.1007/s13280-015-0628-x

**Published:** 2015-02-15

**Authors:** Aimé Jean Messiga, Noura Ziadi, Claire Jouany, Perttu Virkajärvi, Raija Suomela, Sokrat Sinaj, Gilles Bélanger, Ciprian Stroia, Christian Morel

**Affiliations:** 1 Environmental and Resource Studies Program, Trent University, 1600 West Bank Drive, Peterborough, ON K9J 7B8 Canada; 2 Soils and Crops Research and Development Centre, Agriculture and Agri-Food Canada, 2560 Hochelaga Blvd., Quebec, QC G1V 2J3 Canada; 3 INRA UMR1248, Chemin Borde Rouge, BP27, 31326 Castanet Tolosan, France; 4 INPT, UMR AGIR, Université Toulouse, 31029 Toulouse, France; 5 MTT, Maaninka, Halolantie 31 A, 71750 Maaninka, Finland; 6 MTT, Ruukki, Tutkimusasemantie 15, 92400 Ruukki, Finland; 7 Institute for Plant Production Sciences, Agroscope, Route de Duillier 50, Case Postale 1012, 1260 Nyon, Switzerland; 8 Department of Biology and Plant Protection, Faculty of Agriculture, Banat University of Agricultural Sciences and Veterinary Medicine, Calea Aradului 119, 300645 Timisoara, Romania; 9 INRA, UMR 1391 ISPA, 33140 Villenave d’Ornon Cedex, France; 10 Bordeaux Sciences Agro, UMR 1391 ISPA, 33170 Gradignan, France

**Keywords:** Fertilization, Grassland, Long-term field experiments, Phosphorus, Phosphorus budget, Soil test P

## Abstract

We analyzed the linearity of relationships between soil test P (STP) and cumulative phosphorus (P) budget using data from six long-term fertilized grassland sites in four countries: France (Ercé and Gramond), Switzerland (Les Verrières), Canada (Lévis), and Finland (Maaninka and Siikajoki). STP was determined according to existing national guidelines. A linear-plateau model was used to determine the presence of deflection points in the relationships. Deflection points with (*x*, *y*) coordinates were observed everywhere but Maaninka. Above the deflection point, a significant linear relationship was obtained (0.33 < *r* ^2^ < 0.72) at four sites, while below the deflection point, the relationship was not significant, with a negligible rate of STP decrease. The relationship was not linear over the range of STP encountered at most sites, suggesting a need for caution when using the P budget approach to predict STP changes in grasslands, particularly in situations of very low P fertilization. Our study provides insights and description of a tool to improve global P strategies aimed at maintaining STP at levels adequate for grassland production while reducing the risk of P pollution of water.

## Introduction

The difference between fertilizer phosphorus (P) inputs and crop P exports, referred to as the P budget, is often used to assess P management in agroecosystems (Watson et al. 2002; Serrano et al. 2014) and to predict soil P changes over time (Messiga et al. 2012a; Morel et al. 2014). The annual P budget is the key component of global P strategies aimed at lowering the P content of P-saturated soils and monitoring the risks of P pollution of water (Reijneveld et al. 2010). In Finland, negative P budgets are recommended to farmers to reduce the high soil P content of their soils (Saarela et al. 2004; Valkama et al. 2009). In France, the national P budget was reduced to 4.4 kg ha^−1^ year^−1^ in 2006 as a result of reducing P inputs to agricultural soils (Senthilkumar et al. 2012a), while it decreased steadily in Switzerland during the period 1975–2008, to stabilize at 5.5 kg ha^−1^ year^−1^ (Oehl et al. 2002; Spiess 2011). In North America, particularly eastern Canada, less emphasis is placed on the P budget in P fertilizer recommendations; instead the probability of an economic response of crop yield to P fertilizer applications is the central concept of P fertilization strategies (CRAAQ 2010).

Results from long-term experiments with annual field crops (Morel et al. 2000; Tunney et al. 2003) and perennial grass crops (Messiga et al. 2014a; Morel et al. 2014) have shown that soil test P (STP) is closely and positively related to cumulative P budget (*B*_cumP_). This relationship has been shown to vary with location (Blake et al. 2003), soil type (Ciampitti et al. 2011), crop rotation (Oehl et al. 2002), and tillage system (Messiga et al. 2012b). However, for a given site, it is not affected by different rates of N fertilization applied to forage grasses (Messiga et al. 2014a). Studies with annual field crops have shown that a single linear regression can describe changes in STP for both negative and positive P budgets (Blake et al. 2003). For instance, Messiga et al. (2010) grouped data from a maize (*Zea mays* L.) monoculture in France following 7, 12, and 17 years of cultivation and demonstrated that a single linear regression described changes in STP over the three periods. This indicated no or little variation between the parameters of the linear regression in this maize monoculture for a wide range of STP and *B*_cumP_.

The linearity of the STP–*B*_cumP_ relationship over a wide range of STP values as a result of varying P fertilization rates has not yet been studied in long-term and semi-permanent grasslands. In zero-P plots, Ma et al. (2009) reported that STP decreases with time until no further additional decrease can be observed. Under fertilized no-till management systems, Messiga et al. (2012b) observed a more than proportional change in STP for a unit of P budget due to the effect of residual P derived from fertilizer. The P fertilizer applied to grassland is not mixed with the soil and crop residues (Whitehead 2000).

In this study, we hypothesized that in long-term grassland fertilized with varying P rates, the relationship between STP and *B*_cumP_ will not be linear over a wide range of STP values. To investigate this, we assembled a dataset of cumulative P budgets and STP values from six long-term grassland sites with different soil characteristics in four countries (Canada, Finland, France, and Switzerland). The objective of the study was to analyze the linearity of the relationship between STP and *B*_cumP_ in a number of grassland experiments covering a wide range of STP values, obtained as a result of long-term applications of varying P fertilizer rates. To set future national P budget targets that account for both grassland production and water protection, improved and updated knowledge about long-term impacts of current global P management strategies on changes of STP levels is needed.

## Materials and methods

### Site description and fertilization treatments

#### Ercé and Gramond, France

Two experiments on semi-permanent grassland were conducted in the French Pyrenees, France (Table 1). An experiment at Ercé was established in 1999 on an Alfisol (U.S. Soil Taxonomy, Soil Survey Staff 2010) developed on alluvium. An experiment at Gramond was initiated in 1998 on an Inceptisol developed on mica schist. The two grassland sites had a mixture of grass species. The mean annual air temperatures are 12.7 and 11 °C, and the mean annual rainfall rates are 1200 and 960 mm at the Ercé and Gramond sites, respectively. Both experiments consisted of two annual rates of P fertilizer (0 (*P*_0_) and 50 (*P*_50_) kg P ha^−1^) supplied in February as triple super phosphate (TSP, 20 % P) on plots measuring 20 m^2^ (5 m × 4 m) and arranged in four randomized blocks (Table 2). Nitrogen was applied as commercial ammonium nitrate (NH_4_NO_3_) at rates of 100 kg N ha^−1^ in February and 60 kg N ha^−1^ after the first cut. Potassium was added as potassium chloride (KCl) at a rate of 199 kg K ha^−1^ in all treatments once a year in February to maintain an unlimited K supply.

**Table 1:** Location of the six study sites and their clay, sand, and soil carbon content, and soil pH

Sites	Country	Longitude/Latitude	Clay (%)	Sand (%)	Soil carbon (%)	pH
Ercé	France	0°E, 43°N	25.1	25.0	5.7	5.9
Gramond	France	2°E, 44°N	21.4	56.6	3.7	5.5
Les Verrières	Switzerland	7°E, 47°N	28.0	20.9	7.9	5.4
Lévis	Canada	71°W, 46°N	–	–	2.6	5.8
Maaninka	Finland	27°E, 63°N	6.0	66.5	1.6	–
Siikajoki	Finland	25°E, 64°N	8.0	75.3	2.8	–

**Table 2:** Phosphorus (P), nitrogen (N), and potassium (K) fertilization rates, soil sampling depth, study period, and soil test phosphorus analysis at the six study sites

Sites	Country	P rate (kg ha^−1^)	N rate (kg ha^−1^)	K rate (kg ha^−1^)	Soil sampling depth (cm)	Study period	Soil test P
Ercé	France	0, 50	160	199	0–5	1999–2011	Olsen
Gramond	France	0, 50	160	199	0–5	1998–2011	Olsen
Les Verrières	Switzerland	0, 9, 17, 26	25	58	0–10	1992–2008	Ammonium acetate, acetic acid, EDTA
Lévis	Canada	0, 15, 30, 45	120	84	0–15	1998–2006	Mehlich-3
Maaninka	Finland	0, *x* (8–25)	200	50	0–20	2003–2010	Acid ammonium acetate
Siikajoki	Finland	0, *x* (8–25)	200	50	0–20	2003–2010	Acid ammonium acetate

*x* corresponds to annual local recommendations: 25 kg P ha^−1^ in 2003 (establishment year); 10 kg P ha^−1^ in 2004, 2005, and 2006 (established swards); 20 kg P ha^−1^ in 2007 (second establishment year); 8 kg P ha^−1^ in 2008, 2009, 2010, and 2011 (established swards)

#### Les Verrières, Switzerland

A permanent grassland experiment was established in 1993 on a Cambisol at Les Verrières, Switzerland (Table 1), with a mixture of red fescue (*Festuca rubra* L.), common bent (*Agrostis capillaris* L.), and orchardgrass (*Dactylis glomerata* L.). The mean annual air temperature at the site is 5.8 °C, and the mean annual rainfall is 1400 mm. The experiment consisted of four application rates of P fertilizer (0 (*P*_0_), 9 (*P*_9_), 17 (*P*_17_), and 26 (*P*_26_) kg P ha^−1^), in plots measuring 30 m^2^ (10 m × 3 m) arranged in three randomized blocks (Table 2). The P treatments were applied in a single application as TSP (20 % P) in October. Potassium was applied as KCl (58 kg K ha^−1^) in a single application in October, while N was applied at a rate of 25 kg N ha^−1^ as commercial ammonium nitrate in all treatments once a year after the first cut.

#### Lévis, Canada

A grassland experiment was established in 1998 on a Fragihumod at Lévis, Canada (Table 1), with timothy (*Phleum pratense* L. cv. Champ). The mean annual air temperature at that site is 4 °C, and the mean annual rainfall is 692 mm. The experimental design was a split plot, with four application rates of P fertilizer as TSP (20 % P): 0 (*P*_0_), 15 (*P*_15_), 30 (*P*_30_), and 45 (*P*_45_) kg P ha^−1^ assigned to main plots, and four annual applications of N fertilizer (calcium ammonium nitrate): 0 (*N*_0_), 60 (*N*_60_), 120 (*N*_120_), and 180 (*N*_180_) kg N ha^−1^ assigned to subplots (Table 2). Experimental treatments were replicated in four blocks, with individual plots measuring 3.15 m^2^ (2.1 m × 1.5 m). For this study, subplots receiving the four P treatments and 120 kg N ha^−1^ were selected within the experimental setup. From 1999 to 2006, fertilizers were broadcast each year prior to the start of growth in the first week of May. Potassium (KCl) at 84 kg K ha^−1^ was applied simultaneously with P and N to ensure that plant growth was not limited by K.

#### Maaninka and Siikajoki, Finland

Two experiments with sown swards were initiated in 2003 in Finland, at MTT Maaninka on an Entisol and at MTT Siikajoki on a Sulfaquept (Table 1). The mean annual air temperatures are 3.1 and 2.6 °C, and the mean annual rainfall rates are 611 and 538 mm at the Maaninka and Siikajoki sites, respectively. The experiments consisted of either no P fertilizer or annual local recommendations; i.e., 25 kg P ha^−1^ in 2003 (establishment year); 10 kg P ha^−1^ in 2004, 2005, and 2006 (established sward); 20 kg P ha^−1^ in 2007 (second establishment year); and 8 kg P ha^−1^ in 2008, 2009, 2010, and 2011 (established sward) (Table 2). Phosphorus applications were larger in establishment years (2003, 2007) than on established swards to minimize the need for surface P application and, consequently, to decrease P losses in surface run-off, which is the most hazardous form of P losses in Finland (Uusi-Kämppä and Heinonen-Tanski 2008). Phosphorus was applied in a single application in mid-May as TSP (20 % P). Experimental plots were 15 m^2^ (10 m × 1.5 m) at Maaninka and 12 m^2^ (8 m × 1.5 m) at Siikajoki and arranged in a randomized complete block design replicated four times. In the establishment year, a nurse crop (winter barley *Hordeum vulgare* L.) and a mixture (70/30 w/w) of timothy (*Phleum pratense* L. cv. Tuukka; 10 kg ha^−1^) and meadow fescue (*Festuca pratensis* Huds. cv. Ilmari at Maaninka and cv. Kasper at Siikajoki; 15 kg ha^−1^) were seeded. Nitrogen was applied at a rate of 80 kg N ha^−1^ and K at a rate of 50 kg K ha^−1^ in 2003 and 2007, as commercial compound fertilizers. The established swards received 200 kg N ha^−1^ annually divided into two applications, the first in mid-May and the second combined with 35 kg K ha^−1^ immediately after the first cut in mid-June.

### Sample collection and analysis

Soil samples were collected at Ercé and Gramond from 2001 to 2006; at Les Verrières in 1993, 1995, 2001, 2005, and 2008; at Lévis from 2001 to 2006; and at Maaninka and Siikajoki from 2003 to 2010. All soils were collected prior to fertilizer application, air-dried, passed through a 2-mm sieve, and stored at room temperature before analysis. Sampling depth and STP determination were based on existing recommendations in each country: 0–5 cm and Olsen extractable P (*P*_Ol_) for Ercé and Gramond (Olsen et al. 1954); 0–10 cm and a mixture of 0.5 M ammonium acetate, 0.5 M acetic acid, and 0.02 M EDTA extractable P (*P*_AAE_) for Les Verrières (Demaria et al. 2005); 0–15 cm and Mehlich-3 extractable P (*P*_M3_) for Lévis (Mehlich 1984); and 0–20 cm and acidic ammonium acetate extractable P (*P*_AAC_) for Maaninka and Siikajoki (Vuorinen and Mäkitie 1955) (Table 2). All sites were ungrazed, so there was no P return as livestock manure. At each site, the forage crop was harvested twice a year and dry matter (DM) yield was recorded. At Maaninka and Siikajoki, winter barley was harvested in August between 2003 and 2007. Forage P concentrations were determined on samples from Maaninka and Siikajoki following thermal oxidation at 550 °C and dissolution with HCl, and at the other sites using a mixture of sulfuric and selenious acids, as described by Isaac and Johnson (1976). The P removal for an individual cut was calculated by multiplying the forage DM yield by its P concentration. For a given year, annual P removal was obtained as the sum of P removal with the two cuts. The annual P budget was calculated as the difference between P applied as fertilizer and annual P removal, and *B*_cumP_ was calculated by summing up the annual P budgets (Messiga et al. 2012a, b, 2014a).

### Data analysis

Data normality was verified using the Shapiro–Wilk statistic, and homogeneity was verified visually with graphics of the residuals (SAS Institute Inc. 2001). Analysis of variance was conducted for each site using the MIXED procedure of SAS. For *B*_cumP_, *P*_M3_, *P*_Ol_, *P*_AAC_, and *P*_AAE_, the factors considered in the analysis of variance were replicate as random effect, year as repeated measurement, and P application as fixed effect. Differences between least square means (LSMEANS) for all treatment pairs were tested at a significance level of *P* = 0.05. In addition, linear and quadratic effects of P fertilizers on STP were determined for the Les Verrières and Lévis sites using polynomial contrasts. A linear-plateau model (SAS Institute Inc. 2001) was used to fit the STP data to *B*_cumP_. When the linear-plateau model was significant, a deflection point (the point where two straight-line regressions intersect) with (*x*, *y*) coordinates in the range of data of STP plotted against *B*_cumP_ was determined. When the linear-plateau model was not significant, the parameters of the linear regressions between STP and *B*_cumP_ were estimated using the REG procedure of SAS.

## Results and discussion

### Cumulative phosphorus budget

**Fig. 1:**
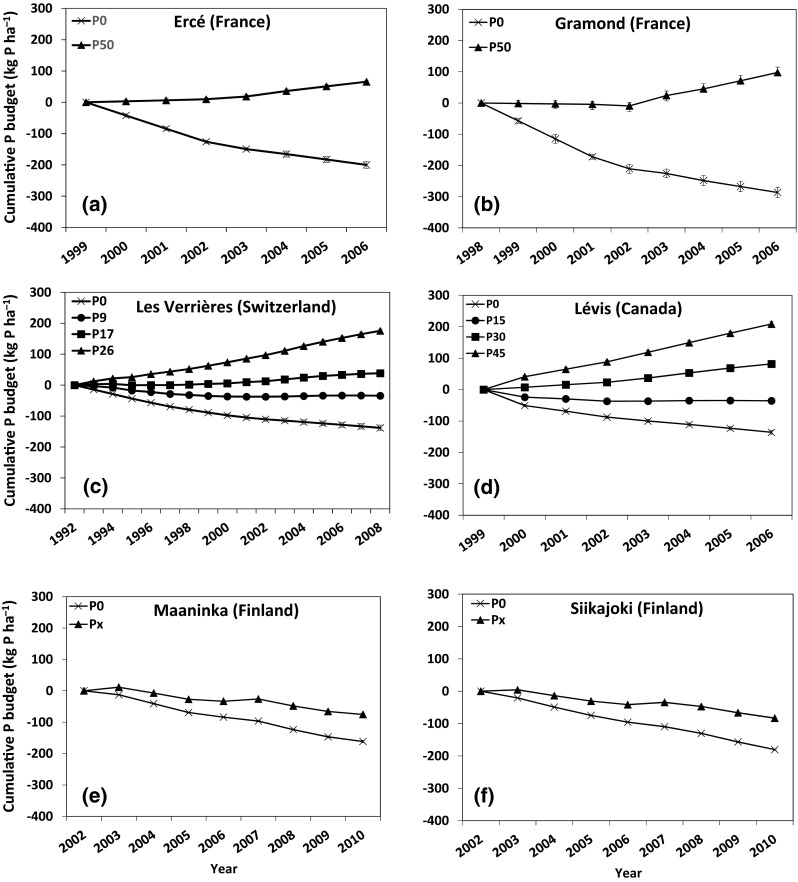
Changes in cumulative P budget at six grassland sites fertilized with various P applications over several years: **a** Ercé, **b** Gramond, **c** Les Verrières, **d** Lévis, **e** Maaninka, and **f** Siikajoki. *Error* *bars* represent ±1 SD of the mean (*n* = 4). *P*_*x*_ corresponds to annual local P application recommendations at the two Finnish sites (8–25 kg P ha^−1^)

The value of *B*_cumP_ varied greatly across sites and P application rates, from a minimum of −287 kg P ha^−1^ at Gramond (Fig. 1b) after nine years without any P application to a maximum of 209 kg P ha^−1^ at Lévis (Fig. 1d) after eight years of P application. At all sites and for the years following the start of the experiments, *B*_cumP_ significantly differed between P application rates (Fig. 1). Negative and decreasing *B*_cumP_ values were associated with annual P applications lower than 15 kg P ha^−1^ (Fig. 1a,b), while positive and increasing *B*_cumP_ values were associated with annual P applications greater than or equal to 26 kg P ha^−1^.

In the two experiments in France, the average annual P budget for the P_50_ treatment was 8 kg P ha^−1^ year^−1^ at Ercé (Fig. 1a) and 11 kg P ha^−1^ year^−1^ at Gramond (Fig. 1b). These values are within the range of annual P budgets observed in 21 French agricultural regions (Senthilkumar et al. 2012b) and comparable to the 8 kg P ha^−1^ year^−1^ average P surplus reported for the European Union (Richards and Dawson 2008; Smit et al. 2009). In France, the national annual P budget in 2006 was 4 kg P ha^−1^ as a result of a national strategy to decrease the total P inflow to soils (Senthilkumar et al. 2012a). Our results indicate that annual applications of less than 50 kg P ha^−1^ are needed to reach this national target on French semi-permanent grasslands.

In the Swiss experiment, the average annual P budget was 10, 2, and −2 kg P ha^−1^ year^−1^ for the *P*_26_, *P*_17_, and *P*_9_ treatments, respectively (Fig. 1c). The current national mean P budget in Switzerland is 5.5 kg P ha^−1^ as a result of direct payments to farmers for environmental programs aimed at decreasing nutrient surpluses (Spiess 2011). Our results indicate that to meet this target, the annual P application to grasslands should be less than 26 kg P ha^−1^.

In the experiment conducted in eastern Canada, the average annual P budgets were 26, 10, and −4.5 for the *P*_45_, *P*_30_, and *P*_15_ treatments, respectively (Fig. 1d). Local recommendations in eastern Canada are based on *P*_M3_ and Mehlich-3 extractable Al (Al_M3_) content. For soils with *P*_M3_ (61–90 kg *P*_M3_ ha^−1^) and Al_M3_ (1100–1600 mg Al_M3_ ha^−1^), i.e., values similar to those of the Lévis site, the recommended P application rate ranges from 24 to 37 kg P ha^−1^ (CRAAQ 2010). Our results suggest that for this fertility class, fertilizer P recommendations will mostly lead to positive *B*_cumP_ values in grassland and, consequently, to the build-up of STP and greater associated risks of P losses. In addition, continuous build-up of STP will decrease the probability of a positive yield response to fertilizer P application.

In the Finnish experiments, the average annual P budget for the fertilized plots was −8 kg P ha^−1^ year^−1^ at Maaninka and −9 kg P ha^−1^ year^−1^ at Siikajoki. This negative *B*_cumP_ with the *P*_*x*_ treatments at both sites (Fig. 1e, f) confirms that P applications corresponding to local recommendations (Valkama et al. 2009) were lower than P removal. The national average application of fertilizer P in Finland was about 30 kg P ha^−1^ in the 1970s and 1980s, but dropped to 12 kg P ha^−1^ during the 1990s in order to reduce the P status of cultivated soils (Saarela et al. 2004). As a consequence of the P fertilization regulations of the national agri-environmental support system since 1995, the national mean P budget decreased from 20 to 8 kg P ha^−1^ year^−1^ during the period 1990–2004 (Organization for Economic Cooperation and Development 2008). The consequence of current Finnish fertilizer P recommendations for grassland, as observed at Maaninka and Siikajoki, is a negative P budget. This is in line with the Helsinki Convention (Helsinki Commission 2005), which has adopted targets to reduce P discharge to waters by 50 % and to improve agricultural practices to maintain or even increase yield levels while lowering nutrient inputs.

### Soil test phosphorus

**Fig. 2:**
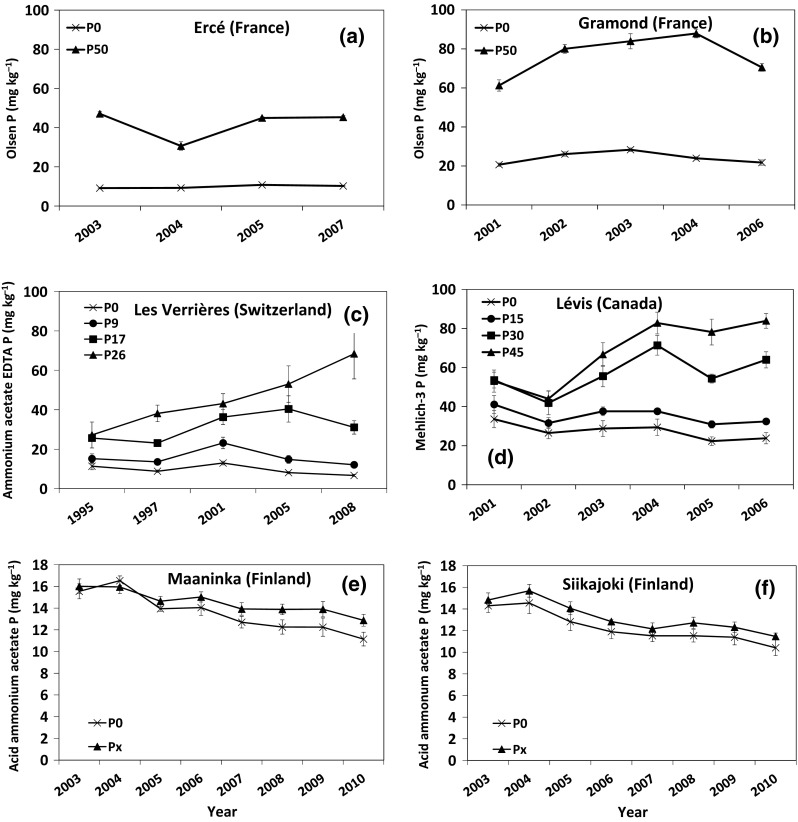
Changes in soil test P at six grassland sites fertilized with various P applications over several years: **a** Ercé, **b** Gramond, **c** Les Verrières, **d** Lévis, **e** Maaninka, and **f** Siikajoki. *Error* *bars* represent ±1 SD of the mean (*n* = 4). *P*_*x*_ corresponds to annual local P application recommendations at the two Finnish sites (8–25 kg P ha^−1^)

The concentration of *P*_Ol_ at Ercé and Gramond was significantly (*P* < 0.001) increased by P application, but the extent of the increase varied with year (P application × year, *P* = 0.002 at Ercé and *P* < 0.001 at Gramond). This positive effect of P application occurred in all years, but the extent of the effect varied slightly from year to year and with no clear trend. The concentration of *P*_Ol_ at Ercé varied from 10.3 mg kg^−1^ in 2003 for the *P*_0_ treatment to 45.3 mg kg^−1^ in 2007 for the *P*_50_ treatment (Fig. 2a), while at Gramond, it varied from 20.6 mg kg^−1^ in 2001 for the *P*_0_ treatment to 87.9 mg kg^−1^ in 2004 for the *P*_50_ treatment (Fig. 2b).

The concentration of *P*_AAE_ at Les Verrières was significantly affected by P application, and the extent of change was affected by year (P application × year, *P* < 0.001). The *P*_AAE_ concentration varied from a minimum of 6.7 mg kg^−1^ in 2008 for the *P*_0_ treatment to a maximum of 68.4 mg kg^−1^ in 2008 for the *P*_26_ treatment (Fig. 2c). It significantly increased with time for the *P*_17_ and *P*_26_ treatments, and decreased slightly with time for the *P*_9_ and *P*_0_ treatments. Overall, *P*_AAE_ values tended to have a quadratic relationship with P application rate.

The concentration of *P*_M3_ at Lévis was significantly influenced by P application (*P* < 0.001), and the extent of change was affected by year (P application × year, *P* < 0.001). The *P*_M3_ concentration varied from a minimum of 23.8 mg kg^−1^ in 2006 for *P*_0_ to a maximum of 83.9 mg kg^−1^ in 2006 for *P*_45_ (Fig. 2d). It significantly increased with time for the P_30_ and P_45_ treatments, and decreased with time for the *P*_0_ and *P*_15_ treatments. Overall, *P*_M3_ values tended to have a linear relationship with P application rate.

The concentration of *P*_AAC_ at Maaninka was significantly influenced by P application (*P* = 0.011) and year (*P* < 0.001). It varied from 15.5 mg kg^−1^ in 2003 to 11.0 mg kg^−1^ in 2010 for the *P*_0_ treatment and from 16.0 mg kg^−1^ in 2003 to 12.8 mg kg^−1^ in 2010 for the *P*_*x*_ treatment (Fig. 2e). The concentration of *P*_AAC_ at Siikajoki was not significantly influenced by P application, but significantly decreased with time (*P* < 0.001) from 14.9 mg kg^−1^ in 2003 to 10.9 mg kg^−1^ in 2010 (Fig. 2f).

Similarly, STP concentrations have been reported in other grassland studies with varying P application rates in France (Jouany et al. 2004), Switzerland (Gallet et al. 2003), Canada (Malhi et al. 2003, 2009), and Finland (Lkhagvasuren et al. 2011), indicating that soil type, soil P content, and management practices encountered in our long-term experiments were representative of grassland management systems in the respective countries.

Differences between the *P*_0_ treatment and P-fertilized plots (i.e., *P*_50_ for Ercé and Gramond, *P*_17_ and *P*_26_ for Les Verrières, and *P*_30_ and *P*_45_ for Lévis) in terms of STP concentrations could be explained by high residual P from fertilizer applied in the P-fertilized plots. The P fertilizer applied to grassland is not mixed with the soil and crop residues, but broadcast on the surface (Whitehead 2000). The transformation of residual P derived from fertilizer to less labile forms might be limited in grassland as this broadcast fertilizer P interacts with plant residues, blocking the sorption sites and, therefore, their adsorption or fixation on the solid phase (Messiga et al. 2012b). Grass stands and residues may alter P availability in fertilized grassland soils by reducing the transformation of soluble P derived from fertilizers to less labile forms by (i) organic anion replacement by H_2_PO_4_ on adsorption sites, (ii) coating of Fe and Al oxides by humus to form a protective cover and reduce P adsorption, and (iii) formation of stable organic complexes with Fe and Al, preventing their reaction with H_2_PO_4_ (Havlin et al. 2014). In addition, the limited transfer of residual P from the enriched upper layer to depleted lower layers due to the low mobility of P also contributes to differences in STP between unfertilized and P-fertilized plots (Messiga et al. 2013).

### Relationship between soil test P and cumulative P budget

**Fig. 3:**
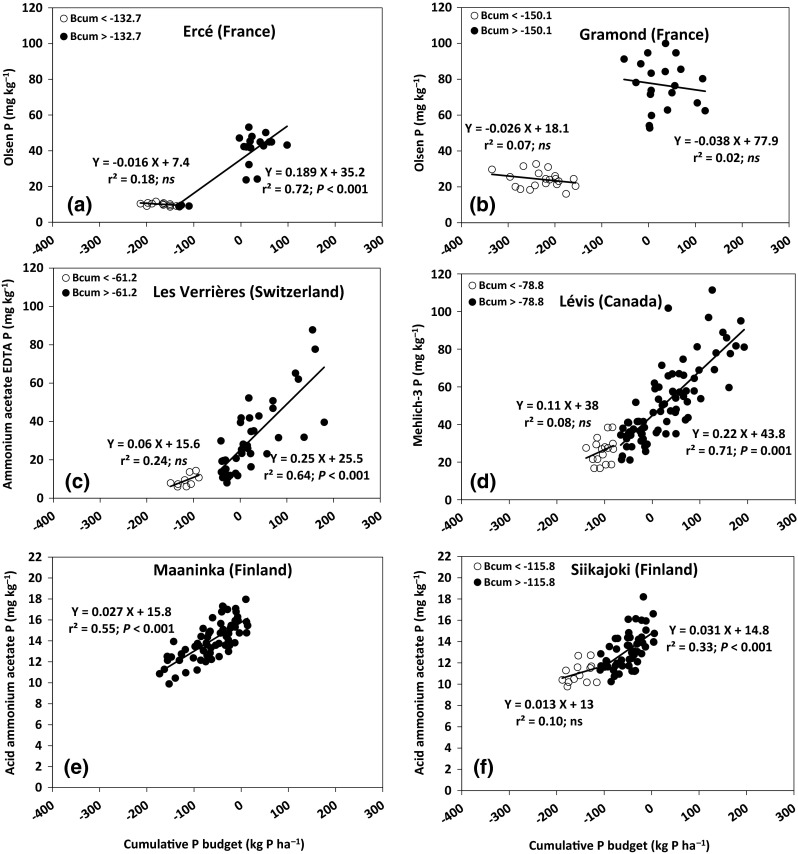
Relationship between soil test P and cumulative P budget (*B*_cumP_) at six grassland sites fertilized with various P applications over several years: **a** Ercé, **b** Gramond, **c** Les Verrières, **d** Lévis, **e** Maaninka, and **f** Siikajoki. *ns* not significant (*P* > 0.05)

The STP generally increased with increasing *B*_cumP_ at all sites. At five of the six sites, however, a deflection point was observed with (*x*, *y*) coordinates of −133 kg P ha^−1^ and 10.1 mg *P*_Ol_ kg^−1^ at Ercé (Fig. 3a); −150 kg P ha^−1^ and 22.0 mg *P*_Ol_ kg^−1^ at Gramond (Fig. 3b); −61 kg P ha^−1^ and 9.3 mg *P*_AAE_ kg^−1^ at Les Verrières (Fig. 3c); −79 kg P ha^−1^ and 26.4 mg P_M3_ kg^−1^ at Lévis (Fig. 3d); and −116 kg P ha^−1^ and 11.3 mg *P*_AAC_ kg^−1^ at Siikajoki (Fig. 3f). At Maaninka, however, no deflection point was observed in the relationship between STP and *B*_cumP_ (Fig. 3e).

Above the deflection point, the fitted linear regression lines were significant (0.33 < *r* ^2^ < 0.72) for Ercé, Les Verrières, Lévis, and Siikajoki, but not for Gramond (Fig. 3). Using the slope of the linear regression above the deflection point at four of the sites and for the whole range of data at Maaninka, we calculated that a change in P budget of 100 kg P ha^−1^ would result in a change of 18.9 mg *P*_Ol_ kg^−1^ at Ercé (Fig. 3a), 25 mg *P*_AAE_ kg^−1^ at Les Verrières (Fig. 3c), 22 mg *P*_M3_ kg^−1^ at Lévis (Fig. 3d), 2.7 mg *P*_AAC_ kg^−1^ at Maaninka (Fig. 3e), and 3.1 mg *P*_AAC_ kg^−1^ at Siikajoki (Fig. 3f). The STP changes for a unit of P budget estimated in this study tended to be greater than values encountered in other field crops under conventionally tilled soils. For example, in a long-term maize monoculture under conventional tillage, Messiga et al. (2010) reported a change of 3.3 mg *P*_Ol_ kg^−1^ and 14 mg *P*_M3_ kg^−1^ for a change in P budget of 100 kg P ha^−1^. Zhang et al. (1995) reported a change of 7 mg *P*_M3_ kg^−1^ in a maize monoculture under conventional tillage in eastern Canada. We could not find values for *P*_AAC_ and *P*_AAE_ in the literature due to a lack of similar studies in the corresponding countries. The greater change rates obtained under grassland indicate the maintenance of high residual P derived from fertilizer. Messiga et al. (2012b) obtained a high change rate of STP for a unit of P budget under no-till systems that translated into a polynomial distribution of STP data plotted against P budget. Soil sampling procedures can also introduce some variability in grassland samples, leading to greater soil test P. For example, shallow soil sampling at Ercé (0–5 cm), Les Verrières (0–10 cm), and, to some extent, Lévis (0–15 cm) resulted in greater change rates compared with Maaninka and Siikajoki (0–20 cm). Differences between extraction methods across the locations could also explain the differences in change rates, but it is difficult to draw conclusions on this point because the effects of this parameter were not tested at our study locations.

Below the deflection point, however, the fitted regression lines were not significant at the sites tested, and there was a slow or negligible STP decline in the absence of P fertilization. The differing relationships between STP and *B*_cumP_ on either side of the deflection point observed at Ercé, Les Verrières, Lévis, and Siikajoki have also been reported in other field crop studies (Webb et al. 1992; Ciampitti et al. 2011). This slow or negligible STP decline in the absence of P fertilization might be explained by the high buffering capacity of the solid phase (Power et al. 2005; Tunney et al. 2010). Ma et al. (2009) reported that in soils with field crops grown for several years without P fertilization, *P*_Ol_ always decreases before stabilizing at a lower limit of 3 mg kg^−1^ despite further net losses of soil P. In a recent study, Messiga et al. (2014b) assessed the depletion of soil P following sequential extractions at several sites in eastern Canada, including the Lévis site, and found that the concentration of *P*_Ol_ by the end of the series of extractions remained constant at 2 mg kg^−1^. At the Rothamsted experimental site, a negative P budget of −509 kg P ha^−1^ was obtained for the period 1901–1974, but the decrease in *P*_Ol_ in the top 23 cm accounted for only 182 kg P ha^−1^ or 36 % of the total P removed in the crops grown in this period (Johnston and Poulton 1977). In another long-term experiment at Saxmundham in Suffolk, UK, the decrease in *P*_Ol_ relative to a negative P budget in the period 1968–1984 accounted for only 10 % of the total P removed in the crops grown in the same period (Johnston et al. 2001). The authors attributed this to plant roots taking up P retained at sites in the soil from which it was not extracted by the Olsen reagent. Another possible explanation for this slow or negligible STP decline with no or little P fertilization might be that plant roots can take up P below the area used for soil sampling. Further studies with more than one sampling depth and sampling at greater depths at different sites would help confirm this hypothesis of P uptake at greater depths in situations of no or low P applications over several years.

Our results confirm the overall positive relationship between STP and *B*_cumP_, but they also indicate that changes in STP can be less in situations of negative *B*_cumP_ and low STP values than in situations of positive *B*_cumP_ and high STP values. Consequently, this relationship between STP and the cumulative P budget should be used with caution when attempts are made at predicting changes in STP. This is particularly true in situations of low STP values. The deflection points reported above provide some values of STP with different soil extractants below which the relationship between STP and *B*_cumP_ should not be used to predict STP changes in grasslands. Above the deflection point, however, the relationship provides a valid estimate of expected changes in STP over time as a function of *B*_cumP_ and could be used as part of a strategy aimed at lowering the P content of P-saturated grassland soils.

## Conclusions

Cumulative P budget was closely related to STP determined according to existing recommendations in each of the four countries: *P*_Ol_ for France (Ercé and Gramond sites), *P*_AAE_ for Switzerland (Les Verrières), *P*_M3_ for Eastern Canada (Lévis), and *P*_AAC_ for Finland (Maaninka and Siikajoki). At five of the six grassland sites, a deflection point was observed in the relationship between STP and *B*_cumP_. Above this deflection point, the relationship was significant and linear at four of the sites and the STP change per unit increase in P budget tended to be greater than values encountered in other field crops under conventionally tilled soils. Below the deflection point, the relationship was not significant and a slow or negligible decrease in STP relative to *B*_cumP_ was observed, probably due to high buffering capacity of the solid phase. These results from long-term grasslands fertilized with varying P rates confirm that the relationship between STP and *B*_cumP_ can deviate for low STP values. The greater STP change per unit increase in P budget observed in most sites indicates that national global P strategies based on P budget approach should be tailored specifically for grasslands. For grassland areas where positive *B*_cumP_ is recommended, detailed soil sampling that capture the P variability along the rooting zone is preferred against shallow soil sampling. In situations with negative *B*_cumP_, soil P dynamics could be altered by high buffering capacity of the solid phase, and therefore the P budget approach should be used with caution for predicting STP changes.
